# Mobile Health Medication Adherence and Blood Pressure Control in Renal Transplant Recipients: A Proof-of-Concept Randomized Controlled Trial

**DOI:** 10.2196/resprot.2633

**Published:** 2013-09-04

**Authors:** John W McGillicuddy, Mathew J Gregoski, Anna K Weiland, Rebecca A Rock, Brenda M Brunner-Jackson, Sachin K Patel, Beje S Thomas, David J Taber, Kenneth D Chavin, Prabhakar K Baliga, Frank A Treiber

**Affiliations:** ^1^Department of SurgeryMedical University of South CarolinaCharleston, SCUnited States; ^2^Technology Applications Center for Healthful LifestylesCollege of NursingMedical University of South CarolinaCharleston, SCUnited States; ^3^Department of NephrologyMedical University of South CarolinaCharleston, SCUnited States; ^4^College of MedicineMedical University of South CarolinaCharleston, SCUnited States

**Keywords:** smartphone, kidney transplantation, medication adherence, mobile health

## Abstract

**Background:**

Mobile phone based programs for kidney transplant recipients are promising tools for improving long-term graft outcomes and better managing comorbidities (eg, hypertension, diabetes). These tools provide an easy to use self-management framework allowing optimal medication adherence that is guided by the patients’ physiological data. This technology is also relatively inexpensive, has an intuitive interface, and provides the capability for real-time personalized feedback to help motivate patient self-efficacy. Automated summary reports of patients’ adherence and blood pressure can easily be uploaded to providers’ networks helping reduce clinical inertia by reducing regimen alteration time.

**Objective:**

The aim of this study was to assess the feasibility, acceptability, and preliminary outcomes of a prototype mobile health (mHealth) medication and blood pressure (BP) self-management system for kidney transplant patients with uncontrolled hypertension.

**Methods:**

A smartphone enabled medication adherence and BP self-management system was developed using a patient and provider centered design. The development framework utilized self-determination theory with iterative stages that were guided and refined based on patient/provider feedback. A 3-month proof-of-concept randomized controlled trial was conducted in 20 hypertensive kidney transplant patients identified as non-adherent to their current medication regimen based on a month long screening using an electronic medication tray. Participants randomized to the mHealth intervention had the reminder functions of their electronic medication tray enabled and received a bluetooth capable BP monitor and a smartphone that received and transmitted encrypted physiological data and delivered reminders to measure BP using text messaging. Controls received standard of care and their adherence continued to be monitored with the medication tray reminders turned off. Providers received weekly summary reports of patient medication adherence and BP readings.

**Results:**

Participation and retention rates were 41/55 (75%) and 31/34 (91%), respectively. The prototype system appears to be safe, highly acceptable, and useful to patients and providers. Compared to the standard care control group (SC), the mHealth intervention group exhibited significant improvements in medication adherence and significant reductions in clinic-measured systolic blood pressures across the monthly evaluations. Physicians made more anti-hypertensive medication adjustments in the mHealth group versus the standard care group (7 adjustments in 5 patients versus 3 adjustments in 3 patients) during the 3-month trial based on the information provided in the weekly reports.

**Conclusions:**

These data support the acceptability and feasibility of the prototype mHealth system. Further trials with larger sample sizes and additional biomarkers (eg, whole blood medication levels) are needed to examine efficacy and effectiveness of the system for improving medication adherence and blood pressure control after kidney transplantation over longer time periods.

**Trial Registration:**

Clinicaltrials.gov NCT01859273; http://clinicaltrials.gov/ct2/show/NCT01859273 (Archived by WebCite at http://www.webcitation.org/6IqfCa3A3).

## Introduction

### Background

Nearly 400,000 people living in the United States suffer with end stage renal disease; of these, approximately 84,000 are currently awaiting kidney transplantation [1,2]. Kidney transplantation is the treatment of choice for eligible patients with end stage renal disease. Kidney transplantation has been shown to offer superior quality of life, improved life expectancy, and better psychosocial functioning, all at less cost than maintenance hemodialysis [3-6].

Despite numerous advances in the medical and surgical care of transplant recipients, significant improvements in long-term graft survival have not been realized. The current 3-year graft survival rate is only 81% [2] and graft half-life is only about 9 years [7]. Remediable factors like poor medication adherence and poor control of comorbid medical conditions negatively impact kidney transplantation outcomes [8-13]. Non-adherence to prescribed medical regimens has been identified as a primary risk factor for graft rejection, graft loss, and death [14-18]. Even in the absence of rejection, non-adherent patients suffer a more rapid loss of renal function over time [17]. While the risk with non-adherence is a continuum, even very small degrees of non-adherence confer a significantly increased risk of graft rejection or graft loss [16,18]. In a recent meta-analysis, researchers found approximately 35% of American kidney recipients demonstrate non-adherent behavior post-transplant [19]. Non-adherence to medication regimens has been shown to develop within just a few weeks of transplantation and its early development increases the risk for persistent poor adherence [16].

Although medication adherence is critical for optimal kidney transplant outcomes, there is a dearth of research examining interventions directed at improving adherence. A recent review of medication non-adherence studies performed in solid organ transplant recipients identified only 12 studies, 7 of which involved kidney transplant recipients [20]. Intervention approaches included patient or primary care provider education and patient-focused motivational, behavioral, or psychological/affective state change. Less than half of the studies observed a significant improvement in adherence to a single medication. Adherence was often evaluated based upon self report and/or medication possession ratio. None of these approaches were completely successful in reaching desired adherence levels.

One approach that has shown promise involved a telephone-delivered adherence self improvement training program coupled with monthly feedback on adherence rates, as measured by an electronic medication event monitoring system (ie, MEMS) [21]. A novel adherence algorithm based on a twice a day dosing schedule was used to calculate an adherence score based on both whether or not the bottle was opened and when relative to the prescribed dosing time it was opened. The intervention group had a significantly higher overall medication adherence score (0.88) than the standard care control group (0.77) over the 6-month randomized controlled trial. However, the impact of the intervention on therapeutic drug levels or physiologic health indices was not assessed.

Following guidelines for user-centered, iterative based, theory-guided development of empirically validated mHealth programs and informed by reviews of prior mHealth interventions [22,23], we conducted semi-structured interviews with renal transplant recipients and their healthcare providers. The objective was to gain an understanding of their awareness, attitudes, and preferences regarding the use of mHealth technology in assisting healthcare delivery and patient self-management. These findings informed the development and administration of a formal survey directed at better understanding kidney transplant recipients’ attitudes, preferences, and utilization of mHealth technology [24]. Of the 99 patients that completed the survey, 90% owned a mobile phone, 52% had access to or owned a smart mobile phone, and the majority was optimistic about the utility of mHealth technology. After being given a demonstration of a prototype mHealth system that was developed based upon the initial interview findings, 90% were receptive to incorporating it into their medical care. Based on findings from this work and additional guidance from patients and healthcare providers, we further refined the prototype mHealth system guided by tenants of self-determination theory to enhance self-efficacy and intrinsic motivation for sustained adherence with medication intake and blood pressure (BP) monitoring [25,26]. BP was selected as the physiologic parameter as the overwhelming majority of kidney transplant recipients have hypertension and many are poorly controlled [27-29].

### Objective

This manuscript describes the results of a proof-of-concept randomized controlled trial (RCT) utilizing this prototype mHealth system in renal transplant recipients with hypertension. The aims of this study were threefold: first, to assess patient and provider acceptability (recruitment and participation rates) and adherence to the protocols; second, to assess the feasibility of using our mHealth system to monitor and enhance medication adherence and BP control; and third, to obtain estimates of variability for the outcome measures and to obtain preliminary indicators of treatment effectiveness, as necessary input for design of a future efficacy/effectiveness RCT.

## Methods

### Study Participants

Study participants were recruited from the Kidney Transplant Clinic at the Medical University of South Carolina (MUSC), Charleston, South Carolina. Potentially eligible study patients were identified through weekly data extractions from the appointment database. Initial inclusion criteria were (1) first time recipient of a functioning solitary kidney transplant performed 3-months earlier, (2) prescribed a total of at least 3 medications for immunosuppression and hypertension, and (3) transplant physician’s assent that patient is able to participate. Exclusion criteria included: (1) inability to self-administer medications, (2) inability to measure own BP, (3) inability to use a mobile phone, (4) history of psychiatric illness or substance abuse, (5) pregnant, lactating or intention of becoming pregnant during the trial, (6) participant in another study, (7) inabilities to speak, hear, or understand English, and (8) poor cellular coverage in their home.

### Prototype mHealth System

**Figure 1:**
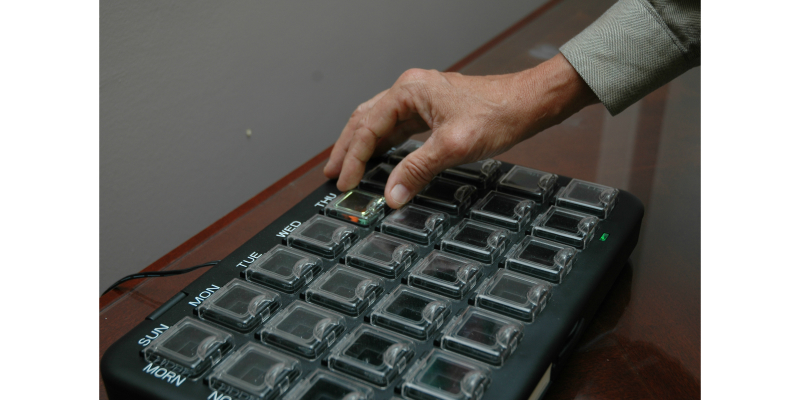
Electronic medication tray (MedMinder).

The prototype mHealth system consisted of a wireless GSM electronic medication tray (MedMinder, Maya, Inc, Needham, MA)[30] (see Figure 1), a wireless bluetooth enabled BP monitor (FORA D15b, Fora Care Inc, Newberry Park, CA)[31], and a smartphone (Droid X, Motorola, Schaumburg, IL)[32]. The medication tray plugs into an ordinary 110V outlet, has 28 compartments (up to 4 doses per day for 7 days), time stamps compartment use, and provides customizable reminder signals. At the prescribed dosing day and time a blinking light from the specific dose compartment was activated. If, after 30 minutes that compartment had not been opened, removed , and returned, a loud chime automatically activated for 30 minutes. If the compartment still had not been opened, an automated reminder phone call or text message was delivered to the subject’s mobile phone. Failure to open the compartment at 90 minutes also generated an automated text message or email that was delivered to the study coordinator. Patients were sent text messages every 3 days as a reminder to measure BP using the FORA device using the standardized resting BP protocol (described below). Blood pressure readings (FORA D15b)[31] were automatically sent via bluetooth to a mobile phone (Motorola Droid X)[32] and from there, via cellular network, to the data repository. No patient names were transmitted and no identifying information was stored on the smartphone. Patients were contacted via the patients’ preferred mode (text, email, or phone) when alerts indicated medication non-adherence, failure to measure BP as scheduled, or that measured BP was outside of threshold ranges established by the patient’s treating physician. In the event that BP readings were outside of safe ranges, the study coordinator was alerted who then contacted the patients and instructed them to obtain additional BP measurements. Persistently unsafe BPs were immediately reported to the treating physician. A weekly summary report, tailored to the treating physician’s preferences, was delivered via email and summarized each subject’s adherence to medication dosing and BP monitoring, as well as breakdown of the BP readings that included systolic and diastolic averages along with the percent of readings that fell into normal and the various stages of hypertension (stage 1 pre-hypertension through stage 2 hypertension). The treating physician made adjustments to the medical regimen as indicated and notified the study coordinator of the changes via email. Any changes made by the treating physician were mirrored in the programming of the medication tray after the study coordinator confirmed with the patient that the changes had been enacted.

### Calculation of Adherence Score

A detailed description of Russell et al’s adherence score calculation is available elsewhere [33]. We employed a modification of her algorithm to allow for dosing schedules other than twice daily. Our subjects were instructed that to be considered fully adherent their medications had to be taken within a 3-hour window centered on the prescribed dosing time. A dose taken within the 3-hour window resulted in a full score for that dosing time; a dose taken outside the 3-hour window but within a 6 hour window resulted in a half score for that dosing time; and missed dose resulted in a score of 0. Each subject was assigned score from 0.0 to 1.0 for each day. The scores for each subject were averaged over each month.

### Identification of Non-Adherent Subjects

Patients who met initial eligibility criteria and provided informed consent were enrolled in a 30-day screening period using the medication tray with its reminder functions disabled. Subjects were given an individual demonstration of how to properly use the medication tray. They were required to demonstrate successful use of the device before completion of their enrollment visit. They worked out tactics with the study coordinator to increase adherence (eg, desired location for device, establishing the protocol as part of daily routine). They received written and oral instructions that to be considered adherent they must take their medications within 90 minutes on either side of the prescribed time. After confirming successful connection with the server, the tray was programmed to accurately reflect the subjects’ medication dosing schedule. At the conclusion of the 1 month screening period our modification of the Russell et al adherence algorithm was used to calculate an adherence score for each subject. In order to construct a non-adherent study population only participants identified as having an adherence score of <0.85 for the month were eligible for randomization into either the mHealth group or the standard care group.

**Figure 2:**
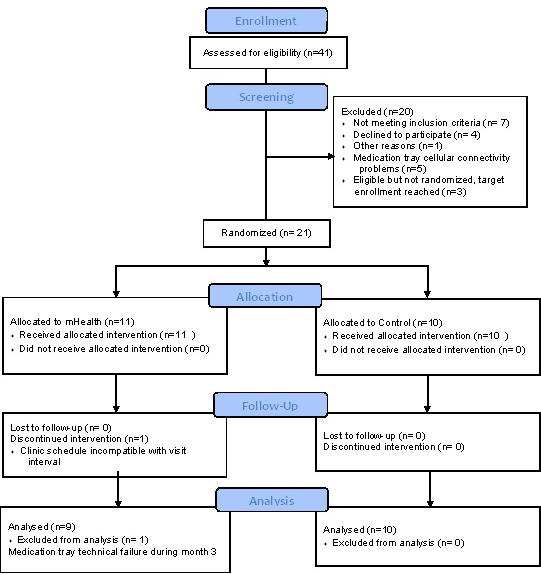
CONSORT flow diagram. A mobile health medication adherence and blood pressure control proof-of-concept trial in renal transplant recipients.

A total of 55 patients were approached for initial recruitment and 41 consented to participate (41/55, 75%)(See Figure 2. CONSORT flow diagram [34]). Of the 14 that declined to participate, 6 stated that they were already adherent with the medication regimen and didn’t need to participate. The other 8 that declined cited concerns with time, travel, and the bulkiness of the medication tray. Each participant provided written informed consent and received gift cards for their participation. A single subject withdrew after consent but before entering the screening period due to concerns about travel related to the study. There were 5 subjects who were removed from the study early after enrollment due to technical issues that were most often related to inadequate cellular phone signal strength at their home. A single subject was removed from the study during the 1-month screening due to graft failure and a return to dialysis. Of the 34 subjects that completed the screening phase, there were 7 with an adherence score >0.85 and were ineligible for randomization. Only 3 of the remaining 27 declined to be randomized into the second phase of the study. From the 3, 2 declined cited time concerns while the third did not explicitly state their reason for withdrawing. There were 3 subjects with adherence scores <0.85 who were not randomized because the study had reached target enrollment and 1 subject was withdrawn after randomization due to difficulties with clinic scheduling. The remaining 20 subjects were randomly assigned to either the mHealth intervention or to standard care. Demographic and transplant-related clinical characteristics of the study participants are summarized in Table 1. The study was approved by the institution’s institutional review board (Clinicaltrials.gov: NCT01859273).

### Standard Care Control Group

The standard care (SC) control group received standard care at the MUSC kidney transplant clinic, which includes visiting the clinic every 4 weeks to 6 weeks depending on the medical indication and time since transplantation. Standard care also includes education on all matters related to post-transplantation medical care and 24-hour phone availability of transplant coordinators. Participants randomized to the SC group continued to use their medication tray, with its reminder functions disabled, for an additional 3 months.

### mHealth Group

The participants randomized to the mHealth group used the prototype mHealth system, described above, for 3 months. The reminder functions of the medication tray were enabled. The subjects in the mHealth group were provided instruction on the use of the FORA device [31] and the smartphone [32] and were required to provide a successful demonstration before completion of their visit. Technical support was available by phone throughout the study. At the conclusion of the study, subjects completed a brief questionnaire assessing their opinions of the mHealth system [35].

### Clinic Resting BP

Evaluations were conducted at pre-intervention and again at months 1, 2, and 3. Patients were seated upright with right arm resting on a table at heart level and a proper cuff size was fitted. The FORA D15 [31] device was used to take the BP measurements. A reading was immediately taken, and after 5 minutes rest, 2 additional readings were taken separated by a 2-minute interval. The average of the last 2 readings was used in the analyses. Subjects in the mHealth group used this same protocol at home for BP self-monitoring. Where a protocol BP was not available, a registered nurse measured clinic BP from the same day was substituted (9 of 76 measurements).

## Results

### Demographic and Clinical Characteristics

The baseline demographic and clinical characteristics of the 2 groups of patients are shown in Table 1. The subjects are representative of the patient population in the MUSC Kidney Transplant Clinic. Although subjects were randomly assigned to treatment condition an independent *t* test t_17_ =3.23, *P*=.002 revealed participants in the SC group (57.6 SE=8.3) were significantly older than mHealth group members (42.4 SE=12.0). However, participants did not differ significantly on months since transplant (*P*=.09) or number of prescribed medications (*P*=.09).

### Acceptability and Feasibility

The acceptability of patients’ participation in either the mHealth or standard care protocol was high with 75% (41/55) of patients approached agreeing to participate in the study. Of the 14 that declined to participate, 6 felt that they were “too adherent” to participate, with the other 8 refusing over concerns that either the electronic medication tray was “too bulky”, that they were “too busy”, or that they would have to travel too much. There was 1 patient who consented but withdrew prior to enrollment due to issues with travel. There were 6 subjects did not complete the lead-in phase, 5 for technical reasons relating to poor cellular signal at their home, and 1 subject was withdrawn due to graft failure necessitating a return to dialysis. Of the 34 subjects that completed the lead-in phase, 7 had adherence scores >0.85 and were ineligible for randomization and 3 were not enrolled due to full enrollment quota (n=20) being reached. Only 3 subjects declined randomization citing time concerns. Of the 21 subjects randomized, 1 was withdrawn for scheduling conflicts; the remainder completed the second phase of the study. There was 1 participant who experienced technical failure of the medication tray during month 3 and was excluded from medication adherence analyses.

The mHealth group reported high overall satisfaction with the mHealth system (average score 4.8/5 point Likert scale: 1= strongly disagree-5 = strongly agree). The mHealth system was easy for the subjects to learn to use (4.7/5) and easy to use in their home (4.8/5). They also found the system useful for medication and health management (4.3/5).

Physicians of the mHealth subjects received weekly reports via email detailing their patients’ adherence rates and average blood pressures. Armed with the information provided, physicians of mHealth patients prescribed more medication changes to anti-hypertensive medications (7 changes in 5 patients) than controls (3 changes in 3 patients).

**Table 1:** Descriptive characteristics of sample.

		mHealth (n=9)	Standard Care (n=10)
**Age in years (SE)**		42.44 (12.04)	57.6 (8.28)
**Ethnicity**			
	Black	6	8
	White	3	1
	Hispanic	0	1
**Gender**			
	Male	4	7
	Female	5	3
**Marital Status**			
	Never Married	4	1
	Married	5	9
**Income**			
	<$15,000	2	2
	$15,000-$29,999	2	4
	$30,000-$49,999	1	2
	$50,000-$74,000	1	1
	No Answer	3	1
**Months since transplant (SE)**		6.33 (2.2)	4.8 (2.6)
**Number of Medications (SE)**		12.6 (2.7)	14.9 (4.5)

### Medication Adherence

#### Screening Period

The average adherence score for all subjects who completed the screening period was 0.63 (SE=0.18) and ranged from high of 0.94 to low of 0.26, those who scored ≥ 0.85 had an average of .90 (SE=0.31) and those who scored < .85 had an average of 0.57 (SE=0.14). The 3 subjects who were eligible to be randomized but refused to continue into the trial had average score of 0.57 (SE=0.12)

#### Trial Phase

The mean monthly adherence rates from pre-intervention screening through study completion by treatment group are presented in Table 2. Medication adherence was examined using a 2 (treatment group: mHealth, SC) x 4 (time: pre-intervention, 1, 2, and 3 months) repeated measures analyses of variance (ANOVA). The repeated-measures ANOVA yielded a significant group by time interaction *F*_3,48_=11.74, *P*<.001, partial η^2^=.42 and a significant main effect for time *F*_3,48_=32.81, *P*<.001, partial η^2^=.673 suggesting that although there was a significant difference across groups at all visits the magnitude of adherence differences increased after baseline. Post-hoc examination of Bonferroni adjusted confidence intervals revealed that the mHealth group did not differ significantly at baseline but displayed significantly higher medication adherence rates compared to the SC during each month following the pre-intervention screening (all *P*s<.05). It is important to note; 1 subject in the mHealth group was omitted due to technical malfunction with the system at month 3.

#### Resting Blood Pressure

**Figure 3:**
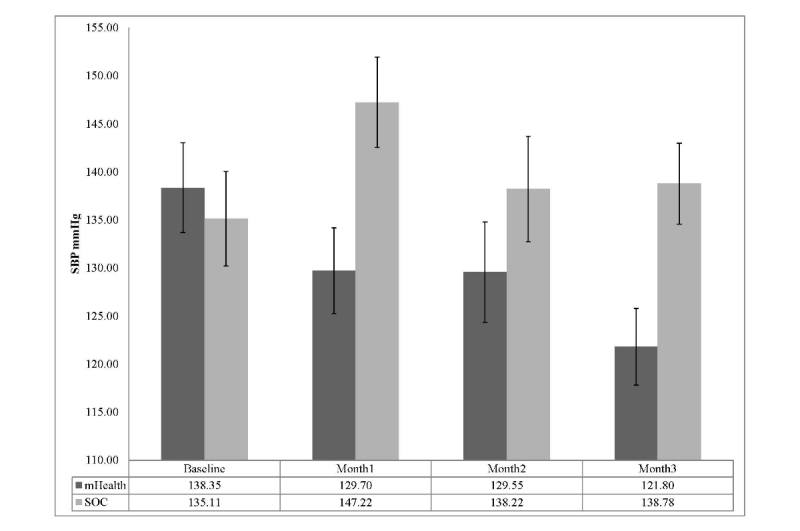
SBP across time by treatment group (mean with Bonferroni 95% CI).

**Figure 4:**
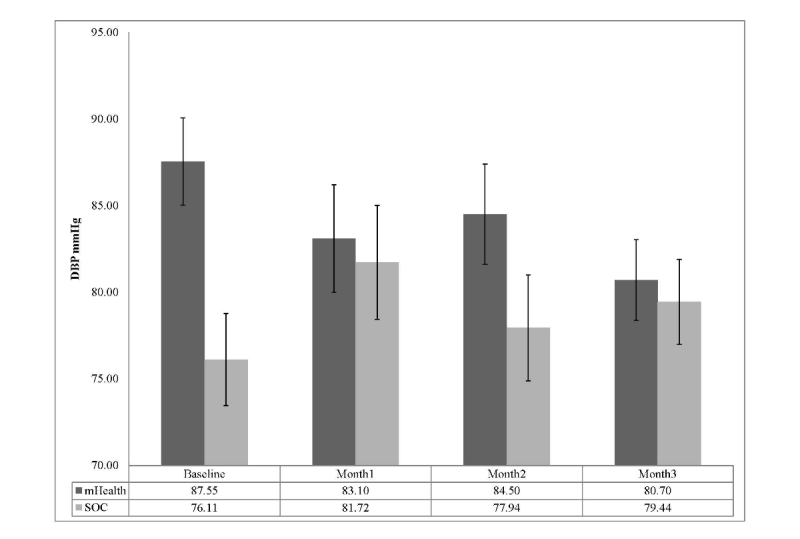
DBP across time by treatment group (mean with Bonferroni 95% CI).

Resting BP was examined using 2 (treatment group: mHealth, SC) by 4 (time: pre-intervention, 1, 2, and 3 months) repeated measures ANOVA. A significant group by time interaction was observed for systolic BP (SBP), *F*_3,51_=4.33, *P*=.009, partial η^2^=.20. Further post-hoc examination of Bonferroni adjusted confidence intervals revealed that the mHealth group demonstrated significantly lower SBPs compared to the SC control group at months 1 and 3. A display of group separation over time is shown in Figure 3. Groups were not significantly different at baseline or month 2. Results for diastolic blood pressure (DBP) also revealed a significant group by time interaction *F*_3,51_=4.58, *P*=.006, partial η^2^=.212. Although groups were randomly assigned based on prescreening adherence, 95% confidence intervals revealed DBP values were significantly different at baseline with those randomized to the mHealth group having an average DBP approximately12mmHg higher. Groups were also significantly different at month three with the mHealth group still revealing significantly higher DBP than SC. Overall, the mHealth group showed a non-linear decline in DBP across months 1-3, while the SC group showed an initial increase at month 1, slight reductions at months 2, and ended with a slight increase at month 3. The pattern of changes across groups is shown in Figure 4.

**Table 2:** **Medication adherence by time across treatment condition** (Bonferroni adjusted 95% confidence intervals[CI]).

Medication Adherence by Time Across Treatment Condition		mHealth (n=9)			Standard Care (n=10)	
	Mean	SE	CI (95%)	Mean	SE	CI (95%)
Baseline	.576	.048	.474-.677	.500	.046	.404-.597
Month 1	.87.4	.046	.777-.970	.533	.043	.442-.625
Month 2	.929	.040	.844-1.014	.587.	038	.507-.668
Month 3	.945	.037	.865-1.025	.574	.036	.498-.650

## Discussion

### Summary

The development of effective, efficient, and non-intrusive approaches to aid kidney transplant recipients self-management and monitoring is critical to success as limited health care provider resources are increasingly taxed by growing demand. Mobile phone based monitoring is an attractive option due to their ubiquity, connectivity, computational power, portability, and relatively low cost [23,36-38]. Recent studies have supported remote monitoring via mobile health (mHealth) technology as an effective and sustainable strategy for facilitating patient-provider communication, increasing adherence to medical regimens, optimizing control of medical conditions, improving health outcomes, and reducing costs in some chronic illnesses [22,23,36,39-44]. While the evidence is mixed at present as to the cost effectiveness of mHealth technology [45], it seems reasonable to hypothesize that it will become so as the cost of the technology decreases and the long-term health benefits are realized. Furthermore as penetrance of the smartphone technology increases, it seems likely that there will be an increasing demand for this type of health care delivery from consumers.

We employed a patient and provider-centered approach to the development of our theory-driven mHealth prototype that allowed us to deliver a system that was highly acceptable to our target population. Feedback elicited prior to study enrollment from key informant interviews and a formal survey study [24] helped inform the study design and facilitated its acceptability and usability. Seventy-five percent (41/55, 75%) of the patients approached agreed to participate in the trial. Nearly half (6/14) of those who declined did so because they felt that they were already highly adherent to their medication regimen. Only 9% (3/34) of the subjects who completed the lead-in screening phase and were eligible for participation in the trial were unwilling to continue. All 20 of the randomized subjects completed the study. The high rates of participation and device utilization suggest that our subjects found the mHealth system to be useful and easy to use, which was confirmed by their responses on the satisfaction and usefulness survey. We intend on conducting focus groups with providers and patients for guidance in further refinement of the mHealth prototype system.

Our study employed a 1-month lead-in to identify patients with poor adherence prior to randomization. Despite all subjects self-reporting high levels of adherence, 78% of those screened were documented to have adherence rates below the cutoff of 0.85, a relatively liberal standard used by Russell et al [21] for patients on immunosuppressant medications. These findings are consistent with the literature that indicates self-report data overestimates objective measures of medication adherence [46] and that medication non-adherence is a significant problem after renal transplantation [19]. Russell et al used a face-to-face cognitive behavioral medication self-management training program to improve objective adherence from 0.72 to 0.88 over a 6-month trial. This improvement is, to date, the most significant reported in the literature for kidney transplant recipients. Our goal was to achieve a comparable improvement using a simpler mHealth-based approach. While Russell et al’s study provided feedback to the patients on a monthly basis there was no mechanism to intervene in real time as the MEMS cap adherence data were only available after being downloaded at the time of the monthly clinic visit. In contrast, the MedMinder device provided an opportunity for real time intervention and feedback. The capacity to intervene shortly after a non-adherent event and to provide timely reinforcement and motivational feedback based upon adherence levels is perhaps the most novel and effective aspect of this trial. Should the improvements in medication adherence prove to be sustainable in longer trials, the rather simple and highly acceptable automated mHealth program has the potential to help resolve what has been a very challenging problem in solid organ transplantation.

In addition to monitoring and encouraging medication adherence, our study investigated the effect of our mHealth prototype on BP control. Blood pressure served both as a surrogate marker of adherence and as a meaningful physiologic indicator of the impact of improved adherence. To our knowledge, no prior study in kidney transplant recipients has simultaneously evaluated the impact of a mHealth intervention on both medication adherence and a relevant physiologic parameter. We observed statistically significant and clinically relevant reductions in clinic based systolic blood pressure (SBP) in the mHealth group compared to the SC control group. Previous BP self-monitoring trials have observed significant BP reductions but the degree of reduction observed in the present trial was far greater (eg, SBP reduction at 3 months: average of -20.3 mmHg versus -8 mmHg across previous RCTs [47-49]. Collectively, the degree of sustained BP reductions observed is quite remarkable given the relative simplicity of the mHealth program compared to the multi-modal face-to-face educational and cognitive behavioral skills based approaches used in previous RCTs [47-50]. We anticipated that our mHealth intervention would lead to more timely adjustments to the subjects’ antihypertensive medication regimens. This was confirmed as mHealth patients were prescribed more anti-hypertensive medication changes (7 changes in 5 patients) than controls (3 changes in 3 patients). For the 5 subjects in the mHealth group who were not prescribed a medication change, BP substantially improved as their adherence increased. This finding can be interpreted as further evidence that, when managing chronic illnesses, the problem is not necessarily that the prescribed medications are not working, but that the patients are not taking them correctly.

These findings must be evaluated within the context of several limitations of the study. First, all subjects were recruited from a single transplant center which may call into question the generalizability of the findings. However, this center is the sole transplant service provider for the State of South Carolina and has a catchment population of over 4.6 million persons that encompass a wide range of ethnic, educational, and socioeconomic backgrounds. Second, that the randomly assigned groups differed significantly in age and adherence prior to the intervention raises questions about the validity of the conclusions. However, within groups age and adherence were not significantly correlated suggesting that age was not responsible for the differences in adherence and BP between our treatment groups. Third, those who chose to participate in the mHealth based RCT might be predisposed to a more positive attitude toward mHealth and thereby introduce a positive bias. That 75% of those approached agreed to participate suggests that a significant bias toward mHealth is unlikely. Fourth, it cannot be assumed that the subjects’ willingness to use the system can be divorced entirely from the fact that the prototype system was freely provided and that they received a small financial reimbursement for their travel costs and time following each clinic evaluation. That many of the subjects asked to continue using the prototype following completion of the trial argues against the financial incentive playing a pivotal role but does not address the question of whether or not they would be as eager to use the mHealth system if it meant spending their own money. Although previous work by this group has documented that nearly 50% of our patient population own smart mobile phones [24], it seems likely that the $45 per month cost of the MedMinder device would prove prohibitive to a large fraction of our patients. Finally, it is important to note that our experiences with the MedMinder devices themselves represent a significant limitation to the broader application of this protocol. We experienced a significant device failure rate of approximately 23%. Without the dedicated attention of our study coordinators and IT personnel, this failure rate would have undoubtedly led to a great deal of patient frustration with the study and poor subject retention.

### Conclusion

To our knowledge, this is the first randomized controlled trial in kidney transplant recipients that has simultaneously examined the use of real time medication reminder and monitoring devices along with wireless measurement of relevant physiological indices to facilitate timely reinforcement based on adherence levels. This study is an early step in our efforts to develop an empirically validated, efficacious, and cost effective mHealth approach dedicated to improving medication adherence, blood pressure control, and minimizing clinical inertia in kidney transplant recipients. In our target population of kidney transplant recipients, our prototype mHealth system was acceptable and resulted in significant improvements in medication adherence and BP control. Although this RCT was not powered to detect an impact on graft function, graft fibrosis, or rejection, the impact on medication adherence and blood pressure control warrant further study. The expected next steps will include a single site efficacy RCT followed by a large-scale multi-site effectiveness RCT with longer follow-up evaluations.

## Abbreviations

BP: blood pressure
DBP: diastolic blood pressure
mHealth: mobile health
MEMS: medication event monitoring system
MUSC: Medical University of South Carolina
RCT: randomized controlled trial
SC: standard care control group
SBP: systolic blood pressure
